# COVID‐19, health care, and abortion exceptionalism in the United States

**DOI:** 10.1363/psrh.12182

**Published:** 2021-08-26

**Authors:** Carole Joffe, Rosalyn Schroeder

**Affiliations:** ^1^ Advancing New Standards in Reproductive Health (ANSIRH) program in the Department of Obstetrics, Gynecology & Reproductive Sciences University of California San Francisco California USA

## Abstract

**Context:**

Few qualitative findings have been published that explore and identify the challenges experienced by independent abortion providers during the COVID‐19 pandemic in the United States (US). In this paper, we explore these themes while expanding the concept of “abortion exceptionalism” beyond its original legal meaning to address the impact of abortion stigma.

**Methods:**

Twenty abortion providers from independent abortion clinics throughout the US South and Midwest participated in semi‐structured interviews in June and July 2020. Interviews explored the challenges of providing abortion care in the wake of the COVID‐19 pandemic and sought to identify how clinics strategized and amended their clinical practices to continue providing abortion care during this time.

**Results:**

All providers we spoke to noted significant challenges to providing abortion care in the early days of COVID‐19. In addition to experiencing the same concerns as other health care institutions, abortion clinics also faced additional, unique burdens that can only be attributed to the politics of abortion exceptionalism. Examples of this abortion exceptionalism include abrupt orders to close clinics, the need to rely on traveling physicians, legislature‐imposed limits on telemedicine, heightened activities of protesters, and non‐evidence‐based regulation of medication abortion.

**Conclusion:**

Despite major challenges and differential treatment, independent abortion clinics in the US persevered to continue to provide abortion care throughout the COVID‐19 pandemic.

## SETTING THE CONTEXT

COVID‐19 has brought significant challenges to virtually all health care services in the United States (US). Patients' needs for in‐person clinical services have had to be weighed against the need to protect both patients and health care providers from contracting or spreading the virus. Health care institutions have made numerous changes to their practices to minimize the threat of infection by both patients and staff. Facilities that provide abortion not only experienced these same challenges but also faced additional and unique challenges. In what follows, we outline the impacts of “abortion exceptionalism,” that is the idea that abortion is regulated both differently and more stringently than other medical procedures that are comparable to abortion in complexity and safety.^1^, ^2^, ^3^, ^4^ This exceptionalism has affected nearly every aspect of abortion care during the pandemic and has placed additional burdens on patients and clinic staff alike.

While the concept of “abortion exceptionalism” was originally used by legal theorists to describe regulatory issues relating to abortion, in this paper, we extend the meaning of the concept to also encompass the highly politicized and stigmatized status of abortion in American society. We argue that this status has significantly impacted abortion care during the pandemic. Yet we also demonstrate that the sheer determination of abortion providers in surmounting these challenges has meant that most abortion care has been able to continue, *albeit* in often quite difficult circumstances.

## SOURCES OF DATA

We base our findings on 20 interviews with abortion providers at independent abortion clinics in the US that were conducted by C. J. in June and July 2020 that explored the impact of COVID‐19 on their clinical practices. We selected these 20 interviewees from a larger sample of 103 respondent clinics from across the country who participated in a rapid‐response quantitative survey that also focused on identifying clinical practices changes that resulted from the COVID‐19 pandemic. We conducted this study with a colleague during April and May of 2020^5^; where appropriate, we also draw on these survey results.

The respondents for both the survey and the follow‐up interviews were recruited via emails sent to the listservs of the Abortion Care Network, the Abortion Clinical Research Network, and via emails sent directly to some known contacts at individual abortion clinics. Altogether, we estimate that ~150–165 clinics were contacted for participation in the study, as membership in the above groups is overlapping.^5^ Throughout this paper as well as the larger study, the term “abortion provider” refers not only to clinicians who perform the abortion procedure but also to clinic directors, managers, and staff at abortion clinics, as it is often the latter who have been charged with troubleshooting the new challenges brought forth by the pandemic.

Although the survey respondents were divided more or less evenly across all regions of the country, for the interviews, we focused primarily on identifying and interviewing those who worked in the Midwest and South, as these regions are regarded to be the most challenging climates for abortion provision. Institutional Review Board of the University of California, San Francisco approved both the survey and follow‐up interviews.

The interviewees included 7 physicians who worked at independent abortion clinics and 13 nonclinical staff, who were a mixture of clinic directors and managers (in small facilities, people typically played both roles). The semi‐structured interviews ranged from 1 to 1.5 h. We analyzed these interviews using both inductive and inductive approaches. Because of the relatively small number of interviewees, we did not use a software program to code the interviews. After conducting a thematic analysis, we organized our results according to the main distinct challenges our respondents reported, highlighting in each case the special role that abortion exceptionalism played.

## FINDINGS

### Clinic closures

We had patients in our waiting room that morning, they were scheduled for their abortions. Then we got a call from our lawyer who told us about the governor's order shutting us down. We had to send them home. *–* Physician in the South.

The quote that opens this section of the paper comes from an abortion‐providing physician and reflects the uncertainty—and even chaos—that descended on abortion clinics located in the 12 “red” states whose governors ordered them to shut down early in the COVID‐19 pandemic on the spurious argument that abortion was not an “essential service.”^6^ These governors made the equally disingenuous argument that abortion care consumed too much “personal protective equipment” (PPE), despite abortion staff wryly pointing out that giving birth consumes far more. Indeed, some observers accused “red state” politicians of cynically using COVID‐19 as an excuse to accomplish their longstanding goal of shuttering abortion clinics entirely.^7^ Ultimately, legal challenges in each state were successful in overturning these temporary abortion bans, which allowed the closed clinics to reopen. However, these closures were costly in several respects. First, interviewees identified closures as the greatest cause of stress among all respondents who operated in the affected “red states.” Second, these ambiguous initial orders were a source of tremendous confusion and anxiety, and in some instances, providers could not ascertain if all methods of abortion care would be prohibited or just those that consumed more PPE. For example, “medication abortion,” which involves the patient taking two types of pills, necessitates less patient‐provider physical contact than “aspiration abortion,” which is the most common form of abortion and requires the provider to be gowned. Finally, clinics wondered if a governor's vague order banning “elective surgeries” applied to abortion care, especially medication abortion care.

As one clinic manager put it early in the pandemic,

We have been on the edge of our seats to find out if the government will shut us down…the anxiety is a torment.

Similarly, a participant in the rapid‐response survey from a Southern state related that

The impact of shuffling patients and telling them that our doors may not be open the next day in the midst of back‐and‐forth legislation was a constant sense of chaos and helplessness.

Of the participating clinics in the rapid‐response survey, more than 70% of those located in states that had declared abortion to be “non‐essential” were forced to cancel or postpone appointments, and in some cases, temporarily closed.^5^

One doctor walked us through the volatile period in which her clinic was forced to abruptly close, only to be reopened briefly before closing again, until they were finally able to remain open:

We had an emergency meeting [after the governor issued the first order to close] to talk about what clinic operations could look like, for example, could we still see medication abortion patients?

And in a subsequent meeting involving a phone call with the clinic's legal counsel:

The conversation was very somber. We had lawyers on the phone, telling us if we did continue seeing patients, ‘we can't guarantee that you might not be criminally charged.’ So there was the possibility of losing my license.

The provider went on to describe how in the process of her clinic being abruptly closed several times, some patients simply fell through the cracks:

There are two patients I am thinking of, I don't know what happened with them. One of them I had seen in the hospital where I also work [a hospital that does not provide abortions]. It was a minor who was pregnant and had some worrisome health conditions. I suggested she come to the clinic the following week. But by then we were closed. The other patient was a homeless woman. She came for her consultation day. [This state requires a separate in‐person consultation prior to the abortion.] But when she was supposed to return for her abortion, her ride didn't show up. She called us, asking if she could come back in the evening. We said come back next week. [By law, the patient's abortion had to be performed by the same doctor who counseled her, and this provider only worked in the clinic one day a week.] And by that next week we were closed.

### Staffing issues and traveling doctors

I have one doctor – and if she gets sick, I'm toast. – Clinic director in New England.

As awareness of COVID‐19 increased in March 2020, health care facilities in general began facing the challenges of sustaining appropriate levels of staffing: many staff experienced childcare issues as schools began to close; some staff members became sick with COVID‐19 or had exposure and therefore were required to quarantine, and others were simply too fearful to come to work. Yet as the quote that opens this section suggests, not only did abortion clinics face all these aforementioned issues but they also had the additional burden, in many instances, of experiencing a chronic shortage of physicians that predated the pandemic. Due to the stigmatization of abortion, many abortion clinics must rely on doctors who fly in from out of town. This reliance on traveling doctors—more common in abortion clinics throughout the South and Midwest than on the coasts—often occurs because few local doctors, even if personally pro‐choice, are willing to provide abortions as they fear sanction from medical colleagues and harassment from abortion opponents.^8^

As various states began to impose travel restrictions across state lines and airlines began to impose their own restrictions, this posed complicated logistical concerns for clinic managers who worried whether scheduled procedures could take place. As one clinic director in the Midwest reported,

I have had so many physicians who had their flights cancelled, unexpectedly, right before travel. Or delayed. Or the airline cancelled their return trip. I issued travel letters to all my doctors, in case they were questioned.

This director went on to say,

I even had a private jet ready to go, if our whole domestic flight system fell apart. So I did have that all lined up. I never had to utilize it though – which is good, it costs a fortune.

Moved by the dedication of her doctors, she lauded the extreme efforts some made to keep their commitments to the clinic: “One of my docs, he used to fly, but now he drives seven and a half hours – each way.” One traveling physician reported that he carried a copy of his medical license in case he was ever questioned as to why he was traveling out of state during a period of travel restrictions.

Overall, nearly 1 in 6 clinics among the 103 clinics in the rapid‐response survey reported that their regularly scheduled clinicians were unable to provide abortion care due to travel restrictions. The greatest impact, predictably, was seen among clinics in the South and Midwest.^5^

### No support people in clinic/minimized comforting of patients

**We** [staff] became their support person…People feel so alone. *–* Clinic director in the South.

Another manifestation of abortion exceptionalism during COVID‐19 focused on ever‐changing clinic practices necessitated by the pandemic. Specifically, certain practice changes undertaken to limit patient‐provider contact meant that the longstanding practice of clinics encouraging patients to have a support person accompany them—typically a partner or a parent—had to be suspended. Additionally, clinic staff felt they could no longer physically touch patients except when it was absolutely necessary in the course of the procedure. While other health care facilities also adopted similar practices in the name of both staff and patient safety, it is arguable that the elimination of these physical support practices could be considered *more consequential* for patients within the abortion context due to the special status of abortion in America as a stigmatized and controversial act.^9^ For example, nearly all abortion clinics in the US—and subsequently their patients—experience the harrowing presence of protesters. Such protesting may occur in front of the clinic entrance, on the path leading up to the clinic, in the surrounding streets, and in‐clinic parking lots,^8^, ^9^, ^10^ and these protesters have been especially problematic during COVID‐19. While pre‐COVID‐19, abortion patients walking through a gauntlet of screaming protesters to enter the clinic have been typically comforted by their support people as well as designated escorts, these support structures were largely dismantled during the pandemic.

Yet support persons' importance to patients goes well beyond the need to counter the presence of protesters. In a context where so many patients have been told that abortion is an immoral or even murderous act—and many have often been subject to frightening misinformation about what an abortion procedure actually consists^9^–having a support person present can be an important source of reassurance and comfort. Moreover, although researchers have shown that patients' “decisional certainty” about their abortion procedure is very high,^11^ patients may still have complex feelings about the circumstances that necessitated their decision and may feel comforted by the presence of a support person.

A clinic director in New England sighed when discussing the loss of support persons in the clinic: “We **loved** having family and friends here, it made it so homey, somehow.” A doctor in the Midwest spoke wistfully of the pre‐COVID‐19 days when her clinic would have designated persons accompany a patient during her abortion:

So we don't have someone holding the patient's hand anymore. That's the sad part, there always used to be a patient‐support person sitting by the patient's head, talking with her throughout the whole thing.

The loss of a support person feels especially acute, according to clinic staff, in those heartbreaking situations where wanted pregnancies must be terminated due to unforeseen circumstances. As a director of a large clinic in the South commiserated:

It's horrible, not allowing somebody to have their support person when they are terminating a pregnancy due to a fetal anomaly and they don't want to be alone. But we do the best we can.

In discussing this matter, another clinic director in the South said simply, “Now **we** have to be their support person.” However, showing support to patients during COVID‐19 is more complicated than it used to be. To ensure patient and staff safety, abortion clinics—like other health care facilities—have tried to minimize physical contact as well as limit exposure time between patients and clinic staff. For this reason, clinics have instituted a practice of instructing their patients with access to cars to wait in the clinic parking lot until their appointment time and fill out required paperwork there. Some clinics also encourage eligible patients to consider having a medication abortion, as it involves less contact with staff. Where legally permitted, abortion clinics have also made use of telemedicine for many pre‐procedure steps, such as taking a patient's medical history, which generally would have occurred inside the clinic. However, in yet another example of abortion exceptionalism, 17 states currently ban the use of telemedicine for abortion even as they allow it for other health care services.^12^

Rational as these clinical practice changes may be and as reassuring as they are to staff fearful of contracting COVID‐19 from patients, many providers interviewed for this study nonetheless see a cost to these measures. A director of a clinic in the South lamented what she felt was being lost with the pandemic‐driven policies of separating staff and patients as much as possible. In her case, the small size of the clinic counseling rooms meant that patients and counselors had to be in different rooms. Frustrated with this situation, she noted: “You need to see the patient's face and body language in order to do good counseling.” Given her counselors' reluctance to use their personal phones to FaceTime with patients due to privacy concerns, she purchased iPads so that patients and staff could Zoom with each other. As she put it, when speaking of the various changes necessitated by COVID‐19, “There are just some things I won't compromise on.”

Along similar lines, several of the doctors interviewed spoke pensively of the “new normal,” which inhibits physical contact with patients after an aspiration abortion. As one said, “You know, now I don't come over to give their hand a squeeze, or give them a hug, if they are receptive to it.” Again, this is not to suggest that it is only abortion providers who must refrain from nonessential physical contact with patients at this time. Rather, it is the special status of abortion in American society that involves such unique features as the necessity to walk through a gauntlet of protesters, and often heightened pressure to keep one's abortion a secret from family and friends,^13^ and the widespread and often terrifying misinformation about abortion procedures that gives a special gravity to these gestures of comfort from abortion providers.

### Protesters and COVID‐19

We had to tell our escorts to stay home. Our protesters are out of control. They don't social distance. They harass our patients in the parking lot and our patients come in in tears. – Clinic manager in the South.

Protesters have always a problem for the abortion‐providing community and have become particularly difficult in the COVID‐19‐era, as the quote opening this section of the paper suggests. Many clinics are heavily reliant on volunteer escorts, whose role it is to shield patients from the protesters that may shout at them or attempt to impede them as they enter the clinic.^2^ However, early in the pandemic escorts were told by clinic staff to stay home as a protective health measure, which left protesters with increased opportunities to harass patients.

One administrator in the Midwest whose clinic has had a particularly long and difficult history with very aggressive protesters spoke with emotion about her current situation:

They're horrible, they're just horrible. Today they had a tent pitched [near to the clinic on public land]. It's a new group, they are pacing the length of the building, where they can see there is a door. They are screaming with bullhorns at the door. We definitely could hear them. They have tried to stop cars in ways they have not before…They don't wear masks, they are not social distancing. They are bringing their kids, and some pregnant women. I hired additional security guards.

However, this administrator was deeply touched when several months after the pandemic began and her escorts were told to stay home that some of the leaders of the escort group contacted her and expressed that they wanted to return.

So I said, you have to follow the same rules as the staff, have your temperature checked, we will do a screening.

A clinic director in the South told of being contacted about a protester by her staff after she had already gone home for the day.

One of our staff was getting ready to lock up and in bounced this man we knew to be a protester. He said ‘I'm here to see if you abortionists are complying with Governor [redacted]‘s orders. What accommodations have you made?’ Ironically, our county has much stricter rules than our governor's. So we had to call the police.

The same director also spoke of an elderly protester:

[He's] around 80, so he thinks nobody will say anything, he parked in our private parking lot with his pro‐life sign on his car, and started taking pictures of the license plates on the patients' cars.

Sometimes it is these abortion opponents who call the police or other authorities to complain about alleged public health violations in the clinic. One clinic director in the South was startled to get a phone call from her local police department with whom she enjoyed a good relationship that had been cultivated over many years.

Apparently the antis [as abortion opponents are widely referred to in the abortion‐providing community] have been calling like crazy about the fact we are open. So she [the policewoman] goes, ‘are you allowed to be open?’ So I told her, abortion is an essential service in [this state] and don't let the antis dictate propaganda to the police!

An administrator in the Midwest complained that “the antis keep calling the Department of Health on us for COVID violations.”^5^

Virtually all interviewees who spoke of protester‐related issues mentioned the negative impact they have on patients, who were already very anxious about a clinic visit due to the risk of COVID‐19. As one clinic director in the South put it,

So in addition to all the tension the poor patient was feeling with, ‘Should I come or not? Can I wait? I'm pregnant and I don't want to be. Now I have all these people yelling at me, they're yelling loud, they're coming close to me, they're not physically distancing.’

This director, like others, spoke of a particular problem of patients being accosted in their cars as they waited in the clinic parking lot and of the frustrations that came with protesters' actions even with a responsive police department.

Our escorts have called police several times, but of course, even if its seven or eight minutes later, the antis will have retreated and there's nothing you can do about it.

In a pattern all too familiar to the abortion clinic community, in the instance reported above, the abortion protesters resumed their harassment of patients once the police had left.

A particularly harrowing instance of recent protester behavior occurred in North Carolina early in the COVID‐19 pandemic. In March 2020 while escorts were still coming to the clinic to assist patients, an unmasked protester approached an elderly escort estimated to be in his 60s or 70s. When the protestor was about 8–10 inches away from the escort, according to an observer, she began to cough without covering her mouth several times in the escort's direction, and tauntingly said “Would be a shame if I was sick”.^14^ Exactly 1 year later, in March 2021, a protestor at this same North Carolina clinic accidentally shot himself in the leg with a gun he had brought to the protest.^15^ This event unnerved not only staff at this clinic but also providers elsewhere, as the abortion‐providing community has already witnessed 13 colleagues killed by anti‐abortion extremists.^9^ While this clinic was not involved in this study, word of these incidents quickly spread among the close‐knit abortion‐providing community.

### Bright spots

People would call us and ask, ‘what do you need?’*—*Clinic owner in the Northeast.

Though we have argued that the COVID‐19 pandemic has exacerbated many difficulties inherent to the field of abortion care—difficulties which are arguably a result of abortion exceptionalism—there have also been some bright spots that have arisen for this field during this period. Foremost among these are the many gestures of local community support that nearly all abortion providers interviewed mentioned.

Providers shared numerous stories of supporters in their communities sending food for the clinic staff and for the community's help in securing personal protective equipment (PPE). The experience of one clinic director in the South with respect to community support was typical. She recounted,

We'd never stocked masks in the quantities we needed once the pandemic hit. People donated some masks to us that they got from their dentists' office. Some of our supporters made masks for us. Even people from other states, who saw our Facebook page, sent masks. ‘We had a step up of financial donations.’

A group of students from a medical school called their local abortion clinic with offers to help and ended up providing childcare for the clinic staff's children who were no longer able to attend school. Similarly, a clinic director in New England spoke of her “champions,” referring to an informal group of well‐resourced people who had taken a special interest in her clinic.

They've been with us since the beginning. They're staunch pro‐choicers. They're embedded in the community, elder statesmen and stateswomen who have been longtime donors.

These “champions” not only wrote checks to provide short‐term support to the clinic but also connected the clinic to individuals who were able to locate sources of hand sanitizer, masks, and face shields.

Although some respondents mentioned the poignancy of abortion providers being excluded from the well‐publicized, COVID‐19‐inspired national proclamations of appreciation for health care workers, many providers drew enormous comfort from the numerous gestures of local support they received. What these various forms of community support suggest highlights a paradox of sorts facing the field of abortion care: partly *because* abortion is so deeply contested and its providers have been so relentlessly targeted that the field has been able to develop such deep bonds with supporters and to a degree arguably untrue in other fields of medicine.^2^

Another bright spot reported by some providers included heightened expressions of appreciation from patients. As the director of a clinic in the South said,

People are so appreciative that we're here! Most everyone has been patient with longer wait times due to a skeleton crew. There's a definite bond between the patients and us‐more so than usual.

## DISCUSSION

As with other health care institutions, abortion clinics faced considerable challenges to provide care during the pandemic. However, unlike other health care institutions abortion clinics also faced additional, unique challenges that can only be attributed to the politics of abortion and not to any credible medical issues. Despite the vivid display of “abortion exceptionalism” by hostile state legislators and in addition to the heightened actions by local protesters, the abortion‐providing community has risen to most of the challenges presented by the COVID‐19 pandemic. More than a year after COVID‐19 first became widely visible, most abortion clinics—due largely to the determination of providers—remain open.

In the long run, perhaps the most positive development for abortion providers during the COVID‐19 pandemic has been the development of new and more efficient protocols and practices. While some of the COVID‐19‐driven changes, such as having patients wait in cars and not allowing support persons inside the clinic, were short‐term measures taken to protect staff and patients, these practices will be stopped as soon as it is safe to do so. As of June 2021, and as vaccination spreads through the population, more clinics are already beginning to relax these practices. However, just as in other areas of health care,^16^ some of the innovations developed in response to the pandemic have proven to be a more efficient way of providing abortion care and these practices will be retained even after COVID‐19 is no longer a significant threat.

The role that medication abortion has played during the pandemic deserves special mention. Shortly after the necessity for new COVID‐19‐era protocols became evident, a group of reproductive health experts issued an evidence‐based report that offered “a sample protocol for increasing [medication abortion] access during a pandemic and beyond.”^17^ ^(p.361)^ The report provided evidence that providers could safely eliminate various in‐clinic tests, such as ultrasound and blood draws, that had been part of the standard protocol for medication abortion care before the pandemic. The authors presented guidelines on how to use alternative means to determine eligibility for medication abortion care. They concluded that:

although [Food and Drug Administration (FDA)]‐imposed restrictions on mifepristone dispensing may require patients to go to the abortion provider or facility to obtain the drug, this protocol would enable every other part of the [medication abortion] process to be implemented without any in‐person encounter.^17^ ^(p361)^

In concert with the release of these new guidelines, some leading national organizations of the reproductive health medical community—the National Abortion Federation, the Abortion Care Network, and the Society of Family Planning—offered webinars for their provider members on how to implement this new protocol.

The most desirable aspect of such a protocol in response to the pandemic was that this model allows patients to forego a visit to the clinic altogether and thus reduces the risk of transmission of COVID‐19 between patients and providers. However, due to prevailing FDA regulations that restrict how mifepristone [the first of two medications used in the standard medication abortion regimen] can be distributed, the mailing of pills directly to patients was not possible during the first year of the pandemic when we conducted interviews for this study. Notably, the long, uneven trajectory of the approval and distribution of mifepristone in the US speaks volumes about the “abortion exceptionalism” that is the theme of this paper.

When the US Food and Drug Administration (FDA) approved the use of mifepristone for early abortion in 2000, the agency imposed strict regulations under a system that came to be known as REMS—“risk evaluation and mitigation strategy”. This was an unusual step for the FDA to have taken, given that the REMS system was designed to address highly dangerous drugs, and a body of evidence already existed showing that mifepristone was very safe. Indeed, less than 3% of FDA‐approved drugs are governed by REMS.^18^ The FDA furthermore required that the dispensation of mifepristone had to involve specific actions, under an aspect of the REMS program called “elements to assure safe use” (ETASU). The most consequential of these ETASU‐mandated actions, particularly during the pandemic, is the requirement that a patient must come in‐person to a hospital, clinic, or doctor's office to receive the drug; it cannot be dispensed in a pharmacy or mailed to a patient's home. Of more than 20,000 FDA‐approved drugs, mifepristone is the only one that has such a requirement.^18^

In light of this in‐person requirement, the abortion‐providing community has devised strategies to make medication abortion more accessible, particularly for those who live far from an abortion clinic. The main way this has been done is for abortion‐seeking patients to schedule a visit at a local, general health care clinic wherein they will undergo any required tests, such as an ultrasound and blood draw, and will then speak with an abortion provider via videoconference. After the abortion provider ascertains the patient's eligibility for a medication abortion care and gives instructions, the mifepristone and misoprostol are offered to the patient.^12^ This method of abortion care via telehealth has been extremely successful in improving abortion access in states where telehealth for abortion is available.

When COVID‐19 raised legitimate fears about provider‐patient contact, it also led to hopes among abortion providers that the in‐person requirement to receive mifepristone could be at least temporarily suspended during the course of the pandemic. While the FDA did temporarily suspend this requirement for other drugs listed under the REMS protocol,^19^ it did not opt to do so for mifepristone. The American Medical Association, the American College of Obstetricians and Gynecologists, a former Commissioner of the FDA, and several senators all called for the FDA lift the mifepristone REMS. However, the FDA refused.

In a July 2020 lawsuit brought forth by several medical groups, as well as the reproductive justice group SisterSong, a federal judge in Maryland ruled that the REMS protocol on mifepristone should be temporarily lifted in order to promote patient safety during the COVID‐19 pandemic. Providers in many states began using mail‐order pharmacies to ship mifepristone and misoprostol directly to the homes of abortion patients. However, the Trump administration responded by immediately appealing this decision to the Supreme Court; in January 2021, the Supreme Court overturned the ruling of the federal judge.^20^

In April 2021, the FDA announced it would not enforce the requirement of in‐person dispensing of mifepristone during the course of the pandemic.^21^ In May 2021, the FDA announced it would review the mifepristone REMS.^22^ These moves undoubtedly reflect the recent change in presidential administrations and may be indicative of a stronger commitment to evidence‐based reproductive health care.

The abortion‐providing community hopes that a sufficient body of evidence will be established that demonstrates how the temporary suspension of these REMS restrictions improved abortion access^23^ and which may ultimately support making this suspension permanent. Several pilot studies conducted prior to the onset of the pandemic under research waivers granted by the FDA have already shown promising results of the safety and effectiveness of a direct‐to‐patient telemedicine model of medication abortion access.^24^ Additionally, a recently completed study in Great Britain also makes the case for the continuance of this policy.^25^ All evidence to‐date has pointed toward high safety and efficacy of allowing mail‐based distribution of mifepristone, and abortion providers and reproductive health advocates are hopeful that continued evidence will lead to the eventual disbanding of the REMS restrictions on medication abortion.

## CONCLUSION

In the midst of so much difficulty, and alongside additional challenges brought on by “abortion exceptionalism,” one positive aspect of the pandemic is that the abortion‐providing community has discovered novel ways of improving abortion access, particularly by eliminating unnecessary medical tests and increasing the use of telemedicine. These innovations will likely be retained where permitted, even after the pandemic abates. However, to a worrisome degree, it seems clear that what practices abortion providers will follow in the future—indeed if they are allowed to legally provide abortion at all—will depend not on scientific evidence, but on politics.

## CONFLICT OF INTEREST

The authors declare no conflicts of interest.
